# Medical Device-Associated* Candida* Infections in a Rural Tertiary Care Teaching Hospital of India

**DOI:** 10.1155/2016/1854673

**Published:** 2016-01-24

**Authors:** Sachin C. Deorukhkar, Santosh Saini

**Affiliations:** Department of Microbiology, Rural Medical College, Pravara Institute of Medical Sciences (Deemed University), Loni, Maharashtra 413736, India

## Abstract

Health care associated infections (HCAIs) add incrementally to the morbidity, mortality, and cost expected of the patient's underlying diseases alone. Approximately, about half all cases of HCAIs are associated with medical devices. As* Candida* medical device-associated infection is highly drug resistant and can lead to serious life-threatening complications, there is a need of continuous surveillance of these infections to initiate preventive and corrective measures. The present study was conducted at a rural tertiary care hospital of India with an aim to evaluate the rate of medical device-associated* Candida* infections. Three commonly encountered medical device-associated infections (MDAI), catheter-associated urinary tract infection (CA-UTI), intravascular catheter-related blood stream infections (CR-BSI), and ventilator-associated pneumonia (VAP), were targeted. The overall rate of MDAI in our hospital was 2.1 per 1000 device days. The rate of* Candida* related CA-UTI and CR-BSI was noted as 1.0 and 0.3, respectively. Untiring efforts taken by team members of Hospital Acquired Infection Control Committee along with maintenance of meticulous hygiene of the hospital and wards may explain the low MDAI rates in our institute. The present surveillance helped us for systematic generation of institutional data regarding MDAI with special reference to role of* Candida* spp.

## 1. Introduction

Health care associated infections (HCAIs) or hospital acquired infections (HAIs) are infections that occur during hospitalization but are neither present nor incubating upon hospital admission. Various factors like increasing incidence of hospitalization, rapid advancement in medical technology, and injudicious use of antibiotics along with better adaptation of microbes to the hospital environment contribute to exponential increase in HCAIs [1]. Even in developed nations, HCAIs concern 5–15% of hospitalized patients and can lead to complications in 25–50% of those admitted in intensive care units (ICUs) [2].

HCAIs add incrementally to the morbidity, mortality, and cost expected of the patient's underlying diseases alone. Additionally, these infections have the potential to severely undermine the superlative effect of the clinician. Therefore, prevention and control of HCAIs are an ever escalating threat which needs to be expeditiously managed.

Approximately about half all cases of HCAIs are associated with medical devices [3]. In recent years, mycotic pathogens are increasingly reported as causes of HCAIs. Fungi, especially* Candida* spp., rank 3rd among various leading cause of catheter-associated infections [4].* Candida* spp. can colonize and form biofilm on most, if not all, medical devices in current use [5]. As* Candida* medical device-associated infection is highly drug resistant and can lead to serious life-threatening complications, there is a need of continuous surveillance of these infections to initiate preventive and corrective measures. A number of published literatures on rate of medical device-associated infections (MDAI) are focused on bacterial organisms but unfortunately till date there is a paucity of data on* Candida* MDAI. Therefore, the present study was conducted at a rural tertiary care hospital of India with an aim to evaluate the rate of medical device-associated* Candida* infections.

## 2. Materials and Methods

The study was conducted in the Department of Microbiology, Rural Medical College and Hospital of Pravara Institute of Medical Sciences (Deemed University), Loni, Maharashtra, India. The protocol of the study was approved by Institutional Ethics Committee.

In this study, the following three commonly encountered medical device-associated infections (MDAI), catheter-associated urinary tract infection (CA-UTI), intravascular catheter-related blood stream infections (CR-BSI), and ventilator-associated pneumonia (VAP), as per the definition of CDC's National Nosocomial Infections Surveillance (NNIS) system criteria, were targeted.

The study was conducted for a period of two years (January 2013 to December 2014) and included patients who were admitted to ICU and different wards for more than 48 h and were exposed to medical devices like urinary catheters, intravascular catheter, and endotracheal tubes.

Laboratory methods employed for diagnosis of CA-UTI, CR-BSI, and VAP were as follows.

### 2.1. CA-UTI

In case of CA-UTI, urine sample was collected aseptically from sampling port of indwelling urinary catheter with sterile syringe and needle. The urine sample was inoculated on blood agar, MacConkey's agar, and Sabouraud dextrose agar (SDA) and incubated at 35°C for 24–48 h.

The patient was labeled as a case of CA-UTI, when he/she developed one or more of the following conditions: temperature (≥38°C), urgency, suprapubic tenderness, presence of yeast cells in Gram stained smear prepared from centrifuged urine sample, and isolation of yeast from urine as a pure growth with colony count >10^4^ colony forming units (CFUs)/mL [6, 7]. In case of bacteriuria, colony count >10^5^ CFU/mL was considered significant.

### 2.2. CR-BSI

The CR-BSI was suspected when a patient with central venous catheter developed fever or other symptoms of sepsis of unknown origin. In such cases, two blood samples were aseptically collected; the first blood sample was obtained from the catheter itself and the second from the other arm. Specimens for blood culture were collected at the time of removal of central venous catheter. The central venous catheter was removed by the treating physician following all aseptic precautions and the distal 2 cm segment of the catheter was collected in sterile capped container with the help of sterile scissor. Inoculated blood culture bottles and catheter tip were immediately transported to microbiology laboratory.

The blood culture samples were processed as per standard microbiological protocols and catheter was processed as per semiquantitative method described by Maki et al. [8]. A colony count >15 CFU/plate was considered significant [8]. Isolation of the same organism from catheter and blood culture indicated CR-BSI.

### 2.3. VAP

VAP was suspected in a patient on mechanical ventilator when there was a development of new fever, cough, and purulent expectoration, supported by radiological evidence of a new or progressive pulmonary infiltrate and leukocytosis [9]. Bronchoalveolar lavage (BAL) and tracheal aspirate collected from these patients were inoculated on blood agar, MacConkey's agar, and SDA. The plates were incubated at 35°C for 24–48 h. A colony count >10^3^ CFU/mL (for BAL) and >10^5^ CFU/mL (for tracheal aspirate) were considered as significant [9].

*Candida* isolates were identified up to species level by assessing the formation of germ tubes, sugar assimilation, and colony color on HiChrome* Candida* agar (Himedia Laboratories Pvt. Ltd., Mumbai, India). Hi*Candida* identification kit (Himedia Laboratories Pvt. Ltd., Mumbai, India) supplemented the species identification. Bacterial isolates were identified as per standard microbiological profile.

MDAI rate was expressed as the number of MDAI per 1000 device days and was calculated using the following formula [9]:

(1) $$\begin{array}{c} \left(\;\frac{Number\text{ of patients developing MDAI}}{\text{Total number of device days}}\;\right)\times 1000. \end{array}$$

## 3. Results

During the study period, a total of 13456 patients were exposed to different types of medical devices for a total duration of 43621 days. Out of these, 93 (0.6%) developed different types of MDAI. Bacterial infection was noted in 57 (0.4%) patients, whereas* Candida* spp. were isolated from 36 (0.2%) cases. The overall rate of MDAI in our hospital was 2.1 per 1000 device days. The overall rates of bacterial and* Candida* MDAI (1000 device days) were 1.3 and 0.8, respectively (Table 1).

During the study period, out of 10621 patients with indwelling urinary catheters, only 80 (0.7%) developed CA-UTI. Bacterial CA-UTI was noted in 47 (0.4%) cases, whereas* Candida* spp. were isolated from 33 (0.3%) suspected cases of CA-UTI. The rate of catheter-associated urinary tract candidiasis per 1000 device days was 1.0 (Table 1).

As shown in Table 1, catheter-related blood stream* Candida* infection was noted in only 0.1% of patients exposed to central venous catheterization. The rate of catheter-related blood stream* Candida* infection per 1000 device days was 0.3. In our study, no case of VAP due to* Candida* spp. was seen.

The spectrum of microorganisms isolated from various types of MDAI is shown in Table 2. Among bacterial pathogens,* E. coli* followed by* Pseudomonas aeruginosa* and* Staphylococcus aureus* were the predominant pathogens.* C. albicans* was isolated from 13 cases, whereas 23 isolates were non-*C. albicans* (NCA) spp. Among* Candida* isolates, predominance of* C. tropicalis* was noted.

As shown in Table 2,* E. coli* followed by* P*.* aeruginosa* and* Enterococcus* spp. were the major bacterial isolates from CA-UTI.* C. tropicalis* was one of the most common* Candida* spp. isolated from CA-UTI (Table 2). A total of 03 (0.1%)* Candida* spp. were isolated from CR-BSI. These included a single isolate each of* C. albicans*,* C. tropicalis,* and* C. parapsilosis* (Table 2).

## 4. Discussion

HCAI is one of the most common adverse, iatrogenic events experienced by hospitalized patients. These infections have occurred since care of patients in hospitals began and health care providers have often been haunted by the maxim: “the patient initially responded to the treatment but later he died of an infection.” In recent years, increasing usage of indwelling medical devices has led to an increase in HCAIs.

In the present study, the overall MDAI rate was found to be 2.1 per 1000 device days, which was higher than the rate reported by Singh et al. [9]. However, the rate of MDAI in our hospital was quite low than that reported in studies of Vonberg et al. [10] and Habibi et al. [11]. The rates of MDAI vary with the category of the hospital and health care unit studied. Untiring efforts taken by team members of Hospital Acquired Infection Control Committee (HAICC) along with maintenance of meticulous hygiene of the hospital and wards may explain the low MDAI rates in our institute. In addition, regular sensitization workshops on hospital infection control, hand hygiene, and universal standard precautions are conducted for health care providers of all cadres.

In the present surveillance, the rate of catheter-associated urinary tract candidiasis per 1000 device days was 1.0. Candiduria is an increasingly prevalent HCAI and is rarely noted as a community acquired infection in a healthy person with structurally normal urinary tract [6]. Approximately about 10–15% of health care associated UTIs are due to* Candida* spp. [7]. Presence of indwelling catheter is one of the important risk factors associated with candiduria [6]. Platt et al. reported* Candida* as the causative agent in 27% of all UTI related to indwelling catheters [12]. Candiduria in the presence of other risk factors can predispose to disseminated infections [7].

The predominance of NCA spp. over* C. albicans* was noted in our study. As per Fischer, the microbiology of candiduria is changing with >50% of isolates now belonging to NCA spp. [13]. NCA spp. are not only better adapted to the urinary tract but also more difficult to eradicate than* C. albicans* [1]. The isolates from NCA spp. often demonstrate reduced susceptibility to commonly used antifungal drugs [1]. Therefore, species identification along with antifungal susceptibility testing aids in proper management of* Candida* infections.

*C. tropicalis* was the most common* Candida* spp. isolated from CA-UTI. This observation is in accordance with that of Jain et al. [7] where* C. tropicalis* was the most frequent cause of candiduria in catheterized ICU patients. In recent years,* C. tropicalis* alone, or in association with other species, is more frequently implicated in human infections [14, 15]. As compared to* C. albicans*,* C. tropicalis* demonstrate reduced susceptibility to fluconazole [14].

In our study, the rate of bacterial CA-UTI 1000 device days was 1.4 with* E. coli* as the major bacterial pathogen.* E. coli* is the most common facultative anaerobic commensal of the gastrointestinal tract, which is in close proximity to the genitourinary tract. In addition, some strains bear surface fimbria notably type I fimbriae, which bind to latex catheters and urothelial cells [16]. Singh et al. reported only one case of CA-UTI caused by* Enterococci* spp. in MICU patient [9]. The rate of CA-UTI in their study was 0.6 per 1000 device days. Habibi et al. reported the CA-UTI rate as 11.3 per 1000 device days [11]. Various other studies have reported the rate of CA-UTI ranging from 1.7 to 30 per 1000 device days [9].

Nosocomial bloodstream infections are one of the important causes of morbidity and mortality in hospitalized patients. In USA, more than 200,000 nosocomial bloodstream infections occur each year [17]. Rates of CR-BSI vary depending on the types of ICU, ranging from 2.9 to 11.3 per 1000 catheter days [18, 19]. In a study conducted in 12 ICUs of 7 Indian hospitals, the CR-BSI rate was 7.92 per 1000 catheter days and excess mortality was 4% [20]. In the present study, the rate of CR-BSI was noted as 0.8 per 1000 catheter days which is slightly higher than that reported by Singh et al. [9].

*Candida* spp. are the commonest cause of disseminated mycoses with reported mortality to be as high as 40–50% [21]. They account for about 15% of total HCAIs and more than 72% of nosocomial mycoses [21].* Candida* spp. are the fourth leading cause of BSI in USA but comparatively less common in Europe [22]. However, the exact prevalence of nosocomial* Candida* BSI in India is not known [23]. In our study, the rate of catheter-related blood stream* Candida* infection per 1000 device days was 0.3. A total of 03 (0.1%)* Candida* spp. were isolated from CR-BSI. These included one isolate each of* C. albicans, C. tropicalis,* and* C. parapsilosis*.* C. parapsilosis* has been described as the second or third most common* Candida* spp. responsible for BSI in Europe, Canada, Asia, and Latin America [24]. The capability of* C. parapsilosis* to form a biofilm on intravascular and prosthetic devices, selective growth in hyperalimentation solution, and colonization of human hand favor its survival and dissemination in hospital environment [25]. Long term use of central venous catheter or indwelling devices is an important risk factor for* C. parapsilosis* infection [25].* C. tropicalis* is the most common cause of nosocomial* Candida* BSI in India [15]. This NCA sp. has ability to develop rapid resistance to fluconazole. It is mostly isolated from ICU patients with prolonged indwelling catheters, receiving broad-spectrum antibiotic therapy or with malignancy [25].* C. tropicalis* is also associated with high mortality rates compared to other* NCA* spp. and* C. albicans* [25]. Mansur et al. [26] and Kaur et al. [27] also reported significant isolation of* NCA* spp. from CR-BSI in their studies.

*Klebsiella pneumoniae*,* E. coli*,* S. aureus,* coagulase negative* Staphylococci,* and* P. aeruginosa* were the bacterial pathogens isolated from CR-BSI. Though the type of nosocomial pathogen varies as per the health care setup, the pathogens commonly associated with HCAI are microorganisms prevalent in the hospital environment [9].

VAP remains an important cause of morbidity and mortality despite advances in antimicrobial therapy, better supportive care modalities, and the use of a wide range of preventive measures [28]. It complicates the course of 8–28% of the patients on mechanical ventilation [28]. In the present study, the rate of VAP per 1000 device days was found to be 3.3, which is quite low in comparison to that reported by other researchers.

*K. pneumoniae*,* E. coli*,* S. aureus,* and* P. aeruginosa* were the bacterial pathogens isolated from VAP cases. In the present study, no case of VAP due to* Candida* spp. was seen. This observation was in agreement with other researchers' like Singh et al. (2010) [9] and Lalwani et al. (2014) [28]. Therefore, it can be stated that VAP may be caused by a wide spectrum of bacterial pathogens but rarely due to mycotic pathogens.

## 5. Conclusion

Although all health care setups have infection control policies and staff takes every precaution to avoid infection, the risk of HAI can never be completely eliminated. Therefore, surveillance of HAI is very important to understand the magnitude of the problem and to initiate intensified preventive measures for improvement in patient care. The present surveillance helped us for systematic generation of institutional data regarding three most important types of MDAI (CR-BSI, CA-UTI, and VAP) with special reference to role of* Candida* spp. To the best of our knowledge, the present study is the first to report the scenario of medical device-associated* Candida* infections from rural part of India.

## Figures and Tables

**Table 1:** Rate of medical device-associated infections.

Type of MDAI (number of cases)	Total number of device days	Number of MDAI detected			Infection rate (per 1000 device days)		
		Bacterial (%)	*Candida* (%)	Total (%)	Bacterial	*Candida *	Total
CA-UTI (10621)	32310	47 (0.4)	33 (0.3)	80 (0.7)	1.4	1.0	2.4
CR-BSI (2632)	10113	06 (0.2)	03 (0.1)	09 (0.3)	0.5	0.3	0.8
VAP (203)	1198	04 (1.9)	—	04 (1.9)	3.3	—	3.3
Total (13456)	43621	57 (0.4)	36 (0.2)	93 (0.6)	1.3	0.8	2.1

**Table 2:** Spectrum of microorganism isolated from medical device associated infections.

Organism	CA-UTI	CR-BSI	VAP	Total
Bacterial isolates (*n* = 57)				
*E. coli*	18	01	01	20
*Klebsiella pneumoniae*	06	02	01	09
*Pseudomonas aeruginosa*	09	01	01	11
*Staphylococcus aureus*	02	01	01	04
Coagulase negative *Staphylococci*	03	01	—	04
*Enterococcus* spp.	09	—	—	09

Total	47	06	04	57

*Candida* spp. (*n* = 36)				
*C. albicans*	12	01	—	13
*C. tropicalis*	14	01	—	15
*C. glabrata*	05	—	—	05
*C. krusei*	02	—	—	02
*C. parapsilosis*	—	01	—	01

Total	33	03	—	36
